# Clustering effects of health risk behavior on mental health and physical activity in Chinese adolescents

**DOI:** 10.1186/s12955-020-01468-z

**Published:** 2020-07-03

**Authors:** Xiangren Yi, Zongyu Liu, Wenzhen Qiao, Xiuye Xie, Nuo Yi, Xiaosheng Dong, Baozhen Wang

**Affiliations:** 1 Department of Sport and Health, School of Physical Education, Shandong University, Jinan, 250061 China; 2 Department of Science and Technology, Shandong Institute of Commerce and Technology, Jinan, 250103 China; 3 Department of Human Performance and Health Education, Western Michigan University, Kalamazoo, MI 49008 USA; 4 Department of Kinesiology, College of Health Science, University of Wisconsin- Milwaukee, Milwaukee, WI 53201 USA; 5 School of Public Health, Shandong University, Jinan, 250061 Shandong China

**Keywords:** Risk behavior, Mental health, Physical activity, Adolescent

## Abstract

**Purpose:**

Risk behaviors are significantly impacting physical and psychological health among adolescents, resulting in a tremendous public health issue. The aim of this study is to examine the association of clustered risk behaviors with mental health and physical activity, and identify to what extent the clustering of various risk behaviors is associated with psychological health and physical activity in Chinese adolescents.

**Methods:**

Students aged 16–18 years, male 16.2 ± 1.03, female 16.3 ± 1.56, were recruited from 30 high schools to complete an online questionnaire in fall semester 2017. A structured questionnaire, 2017 state and local youth risk behavior survey was revised, modified, and translated into Chinese. Five questions were designed to assess physical activity times of the last 7 days. Symptom checklist 90 (SCL-90) was used to investigate the mental health status of the participants. Statistical analyses were done employing chi-square tests, two step cluster analysis, logistic regression.

**Result:**

Results illustrate that girls report a significantly higher mean of being bullied in school, electronically bullied, feeling sad or hopeless, and trying cigarette smoking. Two-step cluster analysis and regression analysis find that alcohol use, smoking and sedentary behavior have significant effect on adolescent health. Logic regression demonstrated that risk behaviors have significantly associated with mental health and physical activity in specific cluster.

**Conclusion:**

This study finds that a specific behavior cluster has significant impact on mental health and physical activity among adolescents. Integrating risk behaviors cluster with factors can be employed to target high-risk adolescents who have poor physical and psychosocial health. The research suggest that more effective and feasible school intervention programs can be designed to promote adolescent health-related behavior in terms of those pathways.

## Instruction

Risk behaviors are significantly impacting physical and psychological health among adolescents, resulting in a tremendous public health issue [1, 2]. Youth Risk behaviors are composed of extensive behaviors during the period of school, such as bullying [3], smoking [4] unhealthy diet [5], insufficient exercise [6] and harmful alcohol use [7], unintentional injuries and violence [8] etc., can cause physical diseases, psychosocial disorders [9–11], and premature illness [12]. Monitoring risk behaviors can assist the timely identification of potential preventive strategies [13]. Studies have demonstrated that the clustering of risk behaviors had a synergistic effect, changing one behavior’s influence on other behaviors [14, 15]. The preventive interventions that focus on simultaneously resolving clustered risk behaviors may be more effective and less costly [9, 13, 15]. Such intervention management depends on the health professional’s knowledge of the clustering and features of a variety of risk behaviors.

It is realized that youth risk behavior clusters based on some theories or theoretical models have necessitated the increasing complication and comprehensiveness of risk behavior interventions research [16–18]. Conservation of resources (COR) theory indicated that risk and resilience issues cluster together and impact health, which may verify particularly valuable in considerate health-related resource caravan passageway [19, 20]. The Lazarus & Folkman’s transactional theory of stress to explain that construction of some personal and social resources (self-esteem, self-efficacy, social support) performed as predictors of perceived stress level [21, 22]. Perceived stress should be associated with potential risk behaviors leading to onset of substance use and related risk behaviors heightened during the school period [23].

Traditional health-related behaviors study concentrated on the clustering of unhealthy diet, physical inactivity, sedentary time, and substance use [17, 24], but now includes more recent behaviors of internet use/screen time [16, 25], video gaming [24, 25] and bullying/being bullied [1, 26], all of which have become increasing physical and psychosocial health risks among adolescents.

Research on physical activity and mental health has been cognizant of various behavioral factors associated with physical fitness [26, 27]. It is critical to identify the behavioral issues that are the most relevant correlates of physical activity. The findings have illustrated that actively encouraging youth to participate in more physical activity will reduce screen time in schools, communities and other settings, and promote psychosocial well-being [28]. Significant positive interactions have been identified among risk behaviors, physical inactivity, overweight and obesity, highlighting the importance of promoting physical activity and discouraging health-related risk behaviors in adolescent preventive intervention programs [17, 28].

Despite evidence showing associations among various risk behaviors, physical activity and mental health [29, 30], and the gender difference on suicidal ideation and its patterns with academic, family, social and health-risk factors as well [31].

little is known about the effects of risk behavior clustering on physical activity and psychological health, especially in China. Presently, among adolescents. The social competence, emotional and behavioral problem, and physical fitness status are serious issues among Chinese adolescents [32]. Therefore, it is essential to explore the interaction of a broad, comprehensive cluster of risk behaviors, physical activity and mental health for Chinese adolescents. The aim of this study is to examine the association of clustered risk behaviors with mental health and physical activity, and identify to what extent the clustering of various risk behaviors is associated with psychological health and physical activity in Chinese adolescents. The anticipated outcome of this study will be the increase of Chinese adolescents’ physical activity and correspondingly the reduction of risky behaviors and the promotion of better psychological health in gender. On the basis of the findings, school intervention programs for health-related behaviors will be designed and developed for Chinese adolescents. This research explores detailed methods that can inform efficient future risk behavior interventions targeting adolescents.

## Methods

### Participants and procedure

Aged 16–18 years, 4630 students were randomly recruited from 30 high schools, to complete an online questionnaire in fall semester 2017. These schools were randomly chosen from 10 districts of Shandong Province, China. All students were assisted with the survey under the guidance of regional administrators, physical education teachers and graduate students. Students completed the questionnaire independently during physical education class. Students were informed that all data were collected voluntarily, anonymously and confidentially and maintained under a password-protected website, to be evaluated and processed only by the direct researchers. Parents and students filled out a consent form. The research was approved by the ethical committees of Shandong University, China.

### Measure

A structured questionnaire was developed based on 2017 State and Local Youth Risk Behavior Survey [8] which was revised, modified, and translated into Chinese.

Reliability of the questionnaire was analyzed by Cronbach’s alpha (α = 0.72). Factor analysis had been performed to evaluate construct validity by the Kaiser-Meyer-Olkin (KMO = 0.81) and Bartlett test (χ2 = 2.2, *p* = 0.00). Five questions were designed to assess physical activity times of the last 7 days, such as PE class, lunchtime, after school, evening, and weekend. Specifically, these five questions are “How often did you engage in strenuous exercise (vigorous play, running, jumping, throwing) in gym class over the past seven days?”, “What have you usually been doing at lunch the past seven days (other than eating lunch)?”, “In the past seven days, how many days have you been physically active after school to play sports, dance or do some vigorous exercise?”, “In the past seven days, how many days in the evening have you been physically active doing exercise, dancing or something strenuous?”, “How many times did you play sports dance or do some strenuous exercise last weekend.”. The reliability is Cronbach’s alpha (α = 0.78). Symptom Checklist 90 (SCL-90) [33] was used to investigate the mental health status of the subjects, including 9 subscales: somatization, obsessive-compulsive symptoms, interpersonal sensitivity, depression, anxiety, hostility, terror, paranoia and psychosis. The reliability and validity of the Chinese version has been tested [34, 35].

### Statistical analysis

Statistical analyses were done employing SPSS version 26 (IBM, Chicago, IL, USA). Descriptive statistics were calculated for all characteristics in term of gender, risk behaviors, mental health and physical activity. The numerical variables were articulated as mean and standard deviation (mean *±* sd.) Chi-square tests were utilized to compare the proportion of screen time, smoking, drinking, alcohol, bullying and sedentary behaviors. Two Step Cluster Analysis (TCA) was employed to analyze groups of adolescents with similar behaviors. Logistic regression was performed to assess the association among clustered behavioral factors, mental health and physical activity variables and to identify odds ratio (OR) and the corresponding 95% confidence intervals (CI).

## Results

### Comparison of risk behaviors

A total of 4630 students participated in this survey, male 2199 (47.5%), female 2431(52.5%), aged 16–18 years, male 16.2 ± 1.03, female 16.3 ± 1.56. Table 1 shows the characteristics of the male and female participants and the comparison of their risk behavior. Results illustrate that girls report a significantly higher mean of being bullied in school (χ2 = 41.78, *p* < 0.01), electronically bullied (χ2 = 74.25, p < 0.01), feeling sad or hopeless (χ2 = 74.25, p < 0.01), and trying cigarette smoking (χ2 = 74.25, *p* < 0.01)*,* but boys have a significantly higher mean in other items. The physical activity is not mean different between boys and girls (Table 1).

**Table 1:** Gender differences in risk behavior, physical activity and mental health

	Male	Female	χ2	P
Watch TV	1.68 **±** 1.35	1.41 **±** 1.05	69.79	0.00
Play video or computer games	2.14 ± 1.56	1.73 ± 1.24	1.06	0.00
Use smart phone	2.68 **±** 1.55	2.59 **±** 1.60	35.48	0.00
Use iPad	3.64 ± 1.37	3.69 **±** 1.33	5.22	0.00
Bullied in school	1.87 **±** 0.33	1.94 **±** 0.23	74.25	0.00
Electronically bullied	1.88 **±** 0.33	1.93 **±** 0.25	40.13	0.00
Feel sad or hopeless	1.81 **±** 0.39	1.86 **±** 0.35	19.49	0.00
Try cigarette smoking	1.76 **±** 0.43	1.92 **±** 0.28	2.21	0.00
Age when first tried cigarette smoking	2.12 **±** 1.93	1.32 **±** 1.10	3.23	0.00
Number of days smoking cigarettes	1.40 **±** 1.22	1.09 **±** 0.56	1.73	0.00
Number of cigarettes each day	1.27 **±** 1.13	1.03 **±** 0.65	1.44	0.00
At least one drink of alcohol	2.53 **±** 2.18	1.71 **±** 1.67	2.87	0.00
Age of first drink of alcohol	2.78 **±** 2.26	1.90 **±** 1.82	2.55	0.00
Ride in a car with somebody drinking alcohol	1.24 ± 0.77	1.11 **±** 0.50	41.78	0.00
At least one drink of alcohol	1.75 **±** 1.67	1.32 **±** 1.19	1.58	0.00
At least exercise 60 min per day	4.16 **±** 2.63	3.91 **±** 2.59	20.2	0.01
Physical education class	2.67 **±** 1.29	2.61 **±** 1.16	26.49	0.00
Sports teams	1.61 **±** 0.90	1.50 **±** 0.79	24.68	0.00
^a^Had a concussion	1.62 **±** 1.07	1.48 **±** 0.96	24.16	0.00

^a^During the past 12 months, how many times did you have a concussion from playing a sport or being physically active?

### Factor analysis

Factor analysis was performed for risk behaviors, both the KMO (0.8) and Bartlett test of sphericity (*p* < 0.001) fit for the requirements (Table 2). Results of factor analysis identified that five behavioral patterns were separated for the individual behaviors. The five factors explained approximately 52.79% of variance of the nineteen items. Table 2 presented factor loadings and explained variance. Five main components were refined, mainly distributing (1) problematic screen time (videogames, smartphone, internet, iPad etc.); (2) drinking of alcohol (underage and binge drinking); (3) smoking (underage and smoke cigars days); (4) bully behaviors (bullying and being bullied); (5) sedentary behavior (insufficient physical exercise, long study time).

**Table 2:** Factor analysis of risk behaviors

	Factor1	Factor 2	Factor 3	Factor4	Factor5
Watch TV	0.72				
Play video or computer games	0.6				
Use smart phone	0.42				
Use iPad	0.38				
At least one drink of alcohol		0.81			
Age of first drink of alcohol		0.72			
Ride in a car with somebody drinking alcohol		0.64			
At least one drink of alcohol		0.61			
Tried cigarette smoking			0.79		
Age when first tried cigarette smoking			−0.79		
Days of cigarettes smoking			0.66		
Number of cigarettes each day			0.65		
Electronically bullied				0.73	
Feel sad or hopeless				0.68	
Bullied in school				0.66	
At least exercise 60 min per day					0.72
Physical education class					0.66
Sports teams					0.57
Had a concussion					0.42
Eigenvalue	4.26	1.83	1.69	1.17	1.08
Variance explained %	4.26	9.63	8.88	6.15	5.66
Cumulative variance explained %	14.62	24.88	35.09	44.52	52.79

KMO = .805 (KMO ≥ .60 means that the result of the factor analysis is acceptable)

Bartlett’s test of sphericity: χ2 = 2.2 (df = 171; *p* = .00)

### Cluster analysis

Two-step Cluster Analysis (TCA) identifies four clusters in risk behaviors that details are presented in Table 3. The regression analysis shows that cluster 1 contains the unhealthiest scores in factor 2 and factor 5 behavioral patterns. Cluster 2 and cluster 3 indicate two high risk behavior patterns in factor 3 and factor 5. The results demonstrate that alcohol use, smoking and sedentary behavior have significant effect on adolescent health. Factor 1 are also relatively healthy behavior patterns, but three clusters are in a medium position. Factor 5 contains two high cluster and one medium cluster, meaning that sedentary behavior significantly affects student health.

**Table 3:** Clusters of risk behavior formed by Two Step Cluster analysis

	Cluster1	Cluster2	Cluster3	Cluster4
Factor1	0.25 (low)	−0.30 (low)	0.44 (medium)	0.43(medium)
Factor 2	0.67 (high)	0.03(normal)	− 0.12 (low)	0.11(low)
Factor 3	0.20 (low)	−0.19 (low)	0.60 (high)	0.04 (normal)
Factor 4	0.33 (low)	0.16 (low)	−0.55 (medium)	0.17 (low)
Factor 5	−0.78 (high)	0.70 (high)	0.55 (medium)	0.20 (low)

### Analysis of risk behavior and mental health within cluster and factor

Logic regression demonstrated the relationship between risk behavior and mental health based on the different variables (Table 4). In somatization, compared with cluster 1 in factor 1, the odds ratios (ORs) and 95% confidence intervals (CIs) were 0.97(0.83–1.13), 1.01(0.91–1.11) and 0.99(0.85–1.16) for cluster 2, cluster 3, cluster 4, respectively. In the hostility category, compared with cluster 3 in factor 5, the ORs and CIs were1. 1.16(1.00–1.33), 12(0.98–1.29), 1.06 (0.90–1.24) for cluster 2,cluster3 and cluster 4. In Photic anxiety, the ORs and CIs were 1.13(0.91–1.40), 1.04(0.76–1.41), 0.99(0.76–1.30) for cluster 2, cluster 3 and cluster 4 in factor 5. In Psychoticism, the ORs and CIs were 1.06(0.88–1.27), 1.31(1.02–1.68), 1.13(0.86–1.50) for cluster 2, cluster 3 and cluster 4 in factor 4, respectively.

**Table 4:** Odd ratios (ORs) of the cluster risk behavior and mental health from ordered logistic model

		F1	F2	F3	F4	F5
Somatization	C1	Ref				
	C2	0.97(0.83–1.13)	0.91(0.84–1.00)*	1.11(0.98–1.25)*	0.96(0.86–1.06)	1.17(1.07–1.26)
	C3	1.01(0.91–1.11)	0.97(0.86–1.10)	1.00(0.84–1.21)	1.03(0.89–1.19)	1.05(0.97–1.14)
	C4	1.01(0.93–1.10)	0.99(0.90–1.08)	0.95(0.81–1.10)	0.89(0.761.04)	1.05(0.96–1.15)***
Obsessive-compulsive	C1	Ref				
	C2	0.97(0.83–1.14)	0.95(0.87–1.03)**	1.06(0.94–1.19)	0.97(0.88–1.08)	1.07(0.99–1.16)
	C3	1.00(0.90–1.11)	1.07(0.95–1.21)	1.03(0.86–1.24)**	1.07(0.93–1.23)	1.10(1.02–1.19)***
	C4	0.97(0.89–1.05)	0.94(0.86–1.03)	1.02(0.88–1.19)	1.01(0.86–1.18)*	1.04(0.95–1.14)
Interpersonal sensitivity	C1	Ref				
	C2	0.83(0.80–1.08)	1.10(0.80–1.11)**	1.03(0.85–1.30)	1.10(0.90–1.21)	0.89(0.80–1.18)
	C3	1.16(0.85–1.30)	1.14(0.89–1.32)	1.02(0.73–1.38)**	1.12(0.96–1.32)**	1.25(0.93–1.70)
	C4	0.92(0.83–1.15)	1.13(0.86–1.21)	1.16(0.80–1.38)	1.00(0.85–1.21)	1.04(0.79–1.57)
Depression	C1	Ref				
	C2	0.98(0.84–1.15)	0.96(0.89–1.05)	1.06(0.94–1.20)*	1.00(0.90–1.11)	1.04(0.96–1.13)
	C3	1.03(0.92–1.14)	1.03(0.911.17)*	0.99(0.82–1.19)	1.11(0.961.28)	1.07(0.98–1.16)**
	C4	0.97(0.89–1.05)	0.98(0.89–1.07)	1.01(0.87–1.18)	1.04(0.88–1.22)	1.05(0.95–1.15)
Anxiety	C1	Ref				
	C2	1.01(0.86–1.18)	0.94(0.87–1.03)*	1.11(0.98–1.26)	1.00(0.90–1.11)	1.05(0.97–1.15)
	C3	1.00(0.90–1.11)	1.04(0.92–1.18)	1.07(0.89–1.28)	1.14(0.99–1.32)**	1.07(0.99–1.16)**
	C4	0.96(0.88–1.04)	0.96(0.88–1.05)	1.01(0.86–1.18)	1.03(0.87–1.21)	1.02(0.93–1.11)
Hostility	C1	Ref				
	C2	1.04(0.79–1.36)	0.94(0.81–1.09)	1.05(0.85–1.30)*	1.03(0.85–1.23)	1.16(1.00–1.33)***
	C3	1.04(0.87–1.23)	1.01(0.82–1.25)**	1.02(0.75–1.39)	1.22(0.95–1.57)	1.12(0.98–1.29)
	C4	0.96(0.83–1.10)	1.01(0.87–1.17)	1.06(0.82–1.38)	1.07(0.81–1.42)	1.06(0.90–1.24)
Photic anxiety	C1	Ref				
	C2	1.00(0.76–1.31)	0.91(0.78–1.05)*	1.13(0.91–1.40)	0.99(0.82–1.19)	1.01(0.88–1.16)
	C3	1.01(0.84–1.20)	1.12(0.91–1.39)	1.04(0.76–1.41)**	1.25(0.98–1.60)	1.13(0.98–1.30)**
	C4	1.01(0.87–1.16)	0.95(0.82–1.11)	0.99(0.76–1.30)	1.04(0.79–1.37)	1.04(0.89–1.22)
Paranoid ideation	C1	Ref				
	C2	1.03(0.79–1.35)	0.96(0.83–1.11)	1.07(0.87–1.33)	1.04(0.87–1.25)	1.06(0.92–1.22)
	C3	1.07(0.90–1.28)	1.09(0.88–1.35)	0.91(0.66–1.23)	1.36(1.06–1.74)***	1.17(1.01–1.34)***
	C4	0.96(0.83–1.11)	1.02(0.88–1.19)	1.02(0.78–1.33)	1.19(0.90–1.57)	1.01(0.86–1.18)
Psychoticism	C1	Ref				
	C2	1.02(0.78–1.33)	0.94(0.81–1.09)*	1.12(0.91–1.39)**	1.06(0.88–1.27)	1.06(0.92–1.22)
	C3	1.03(0.87–1.23)	1.02(0.82–1.26)	0.97(0.71–1.32)	1.31(1.02–1.68)**	1.12(0.98–1.29)
	C4	0.97(0.841.12)	1.00(0.85–1.16)	1.02(0.78–1.32)	1.13(0.86–1.50)	1.02(0.87–1.19)
Additional items	C1	Ref				
	C2	1.16(0.89–1.52)	0.93(0.81–1.08)	1.10(0.89–1.35)*	0.98(0.82–1.18)	1.09(0.94–1.25)
	C3	1.07(0.89–1.27)	1.06(0.85–1.30)*	1.04(0.76–1.41)	1.13(0.89–1.45)*	1.17(1.02–1.35)**
	C4	0.99(0.86–1.14)	0.97(0.83–1.13)	1.01(0.78–1.31)	1.05(0.80–1.38)	1.07(0.92–1.25)

*p* < 0.05*

*p* < 0.01**

*p* < 0.001 ***

In the first model, we compared participants with physical education class times of the ORs and 95% CIs were 0.75(0.57–1.00), 0.86(0.72–1.03) and 0.88(0.76–1.02) (Table 5). The result show that screen time is significantly associated with PE class time in cluster 2. In the second model, we adjusted a variable and added physical education class time. The result show that cluster 2 and cluster 3 in factor 1(screen time) are very significantly associated with at lunch time. In the third model, we added physical activity at lunch time, cluster 4 in factor 4 (bully behaviors) is significantly associated with after school. In the fourth model, the results show that cluster 4 in factor 3 (smoking) and factor 4 (bully behaviors) are significantly associated with evening time. In the fifth model, the result is almost same as the fourth model.

**Table 5:** Odd ratios (ORS of the cluster risk behavior and physical activity from ordered logic model)

		F1	F2	F3	F4	F5
Model^1^	C1	Ref				
	C2	0.75(0.57–1.00)***	0.99(0.85–1.57)	1.06(0.85–1.33)	0.74(1.08–1.32)	0.99(0.85–1.15)
	C3	0.86(0.72–1.03)	0.75(0.60–0.95)	1.07(0.76–1.50)	0.98(0.76–1.27)	0.82(0.71–0.95)**
	C4	0.88(0.76–1.02)	1.03(0.87–1.20)	1.34(1.01–1.78)	0.93(0.7–1.24	1.00(0.85–1.18)
Model ^2^	C1	Ref				
	C2	1.51 (1.13–2.02)**	1.00(0.86–1.16)	1.02(0.82–1.27)	0.89(0.74–1.08)	0.95(0.81–1.10)
	C3	1.30(1.08–1.57)**	0.77(0.62–0.97)**	1.03(0.74–1.43)	0.83(0.63–1.08)	1.08(0.93–1.25)
	C4	1.12(0.96–1.31)	1.02(0.87–1.20)	1.37(1.04–1.81)**	0.88(0.66–1.18)	1.01(0.85–1.20)
Model ^3^	C1	Ref				
	C2	1.13(0.84–1.51)	1.02(0.87–1.2)	0.91(0.72–1.15)	0.96(0.79–1.16)	0.93(0.80–1.09)
	C3	1.11(0.92–1.34)	0.98(0.78–1.22)	1.23(0.88–1.72)	0.84(0.65–1.10)	1.10(0.95–1.28)
	C4	0.99(0.85–1.16)	0.99(0.84–1.17)	1.17(0.88–1.56)	0.73(0.54–0.99)**	1.07(0.90–1.27)
Model ^4^	C1	Ref				
	C2	1.18(0.89–1.58)*	1.03(0.88–1.20)	1.1(0.87–1.38)	0.98(0.81–1.19)	1.01(0.86–1.17)
	C3	0.94(0.78–1.14)	0.87(0.69–1.09)	1.19(0.85–1.67)	1.09(0.84–1.42)	1.13(0.97–1.30)
	C4	0.9(0.77–1.05)	0.98(0.83–1.15)	1.06(0.79–1.40)**	0.85(0.63–1.15)**	0.97(0.82–1.116)
Model^5^	C1	Ref				
	C2	1.03(0.77–1.39)	1.04(0.89–1.23)	1.12(0.89–1.41)	1.05(0.87–1.28)	1.01(0.86–1.18)
	C3	0.86(0.73–1.07)	1.02(0.81–1.28)	1.15(0.82–1.62)	1.04(0.80–1.36)	1.22(1.05–1.42)*
	C4	0.91(0.78–1.1)	0.94(0.79–1.11)	1.20(0.91–1.60)**	0.84(0.62–1.14)**	1.03(0.87–1.22)

Logic regression showed the relationship between physical activity and risk behavior in the 6 models in terms of the different adjusted variables. We added an adjusted variable for each model, respectively, except for model 1

*p* < 0.05*

*p* < 0.01**

*p* < 0.001 ***

## Discussion

The purpose of this study was to examine the association of clustered risk behaviors with adolescent mental health and physical activity, and identify to what extent the clustering of various risk behaviors is associated with psychological health and physical activity in Chinese adolescents. The research evaluates a broad spectrum of risk behaviors associated with mental health and physical activity in boys and girls. The results showed that risk behavior may have multiple clustering effects on mental health, which consistent with the transactional theory of stress and coping risk and resilience issues [19]. Under different variables, the relationship between risk behavior and mental health is different. In reference to cluster and factor analysis, it was found that specific risk behavior is related to specific mental disorders, including psychosomatic, obsessive-compulsive, intrinsic sensitivity, anxiety, hostility, psychosis and other mental disorders. This study illustrates the significant difference of risk health behaviors and mental health issues, except for physical activity, between boys and girls. Consistent with previous studies, boys are more likely to participate in risk behavior than girls. Girls are more likely than boys to have feelings of academic pressure, and boy are more likely to participate in smoking, alcohol abuse. The girls are more likely to be bullied in school, bullied electronically, feel sad or hopeless, and to try smoking [32].

Factor analysis identified five main factor patterns in this study:1) problematic screen time use; 2) use of alcohol; 3) smoking; 4) bully behavior; 5) sedentary behavior. Four clusters in five factors were distinguished in that four clusters (1–4) in factor 1 (screen time) and factor 4 (bully) have relatively normal all-behavioral pattern scores, which means that screen time and bullying/electronically bullied Chinese students is not a very serious problem, mainly because high school students are not allowed to bring cell phones/iPads on campus, which also reduced electrically bullied reduced greatly*.* This study demonstrate that alcohol use, smoking and sedentary behavior have significant effects on adolescent health. Factor 5 contains two high cluster and one medium cluster, which means that sedentary behavior significantly affects adolescent health in China.

Regarding problematic screen time use and mental health, this study found that the screen time is not associated with psychological health problems after monitoring for its obsessive component in the regression analyses, as is consistent with previous study [13, 28, 36]. This illustrates the position of multi-behavioral factors and clustering analyses when considering behavioral and mental health risk in adolescents as well as the hazard of overemphasizing effects when exploring these associations in a solitary study [36]. However, some evidences associate screen time with mental health problems [31, 36–41]. Those studies indicate a U-shaped relationship between screen time behaviors and psychological problems [40, 41], in which a certain amount of time gone through a certain status (duration/intensity), the screen time behavior becomes problematic and associates with decreased mental health. Additionally, new smart phones/games/websites are developing rapidly and make it hard to properly scrutinize the relationship of screen-time behavior to mental health problems.

The association between use of alcohol and psychological problems is quite controversial. Some studies have indicated no direct relationship with light, moderate and heavy drinkers [42] who have associated with depression and anxiety. Some studies found that alcohol abstainers may develop higher levels of anxiety and depression compared to those who are not alcohol abstainers [43]. However, it is difficult to elucidate these results to sustain the beneficial impacts of moderate alcohol consumption. Sociodemographic characteristics (e.g., gender, age, education, working status), tobacco, and BMI were considered as control variables, but the U-shaped relation may not illustrate the association between alcohol use and psychological problems. In a different analysis, the positive association between moderate alcohol consumption and psychological problems may be impacted by sociodemographic features or unhealthy lifestyle and not triggered by the alcohol use itself [44]. The present study shows that alcohol consumption influences psychological problems in specific factor-clusters, such as obsessive-compulsive disorder, interpersonal sensitivity, hostility, depression, anxiety. Although previous evidence regarding alcohol use and psychological problems was diverse [45]. This study presumed that more frequent alcohol consumption would cause mental health problems. Our proposition was tested only in Chinese adolescents. Some research has demonstrated that students drink more frequently and present greater psychological well-being, which may be explained by those students drinking alcohol on a regular basis rather than binge-drinking, or displaying other sociodemographic features, such as higher socioeconomic status, more social activity and better personal or familial relationships [46].

Tobacco use is recognized as a risk behavior for mental health [46, 47]. Our assumption regarding the association of smoking with mental health was supported in this study. Smoking in Chinese students was a positive predictor of psychological problems related to photic anxiety, psychoticism, obsessive- compulsive disorder, and interpersonal sensitivity. Some studies have found that people who quit smoking undergo a significant decline in symptoms of stress, depression and anxiety, and a rise in life satisfaction and emotional well-being, which contrasts with continuing smokers [48, 49]. Tobacco use is associated with symptoms of depression during early adolescence [50]. Youth smokers with depression and anxiety may develop a tobacco addiction in early adulthood [51]. Further promoting smoking cessation programs might assist more adolescents to stop tobacco use and thereby not only enhance physical health, but also their psychological well-being [52].

Evidence demonstrates that bullying has become a serious and pervasive problem**,** and bullied teenagers are more vulnerable to psychological harm in their lives [53]. The experiences of being bullied or bullying others are associated with psychological problems in adolescence [13]. This study shows that bullying behaviors are significantly associated with interpersonal sensitivity, paranoid ideation, psychoticism, and anxiety in Chinese adolescents. The previous study demonstrated that children and young people who have been bullied will often suffer from long-term mental health problems, such as social isolation, depression, loneliness, anxiety, and suicidal ideation. Perpetrators of bullying also experience a heightened risk of psychological problems such as depression, anxiety, and antisocial behavior [54]. In addition, recent rapid advances in electronic communication technologies also greatly increase the likelihood of this factor emerging. Determining the factors related to bullying is helpful in formulating effective intervention measures for school adolescent bullying [55]. Overall, adolescents with mental health struggles have been recognized as victims of bullying more often, are more frequent game players and compulsive internet users and have more habits of physical inactivity, unhealthy diet and smoking than those without psychosocial problems [56].

Chinese high school students have serious sedentary behavior because they face heavy schoolwork burdens which require total sitting times of 12–13 h or more. If we count typical student daily activities including 8-h class study, 8-h sleep, 1.5-h three meals, 1-h break time, then the remaining 5.5 h are spent in sedentary behavior [57]. We compare to South Koreans [58] at 8.9 h/day, Americans [53] at 7.5 h/day, and Canadians [59] at 9.1 h/day, which includes all their daily sedentary behavior: homework, watching TV, internet, parties etc. Most of the sedentary behavior of Chinese adolescents is education-related, including after-school homework and tutoring, reflecting a critical education problem in China. Homework and tutoring occupy most of the sitting time that seems to reflect characteristic of Chinese adolescents*.* In contrast, screen-related sedentary behavior comprises most of the sitting time of foreign children and adolescents [60].

This study suggests that sedentary behavior is associated with somatization, obsessive-compulsive disorder, depression, anxiety, hostility, photic anxiety, and paranoid ideation. Consistent with previous research, reduced sedentary behavior and increased physical activity may mitigate various psychological problems, including low self-esteem, depression, inappropriate social behaviors and poor emotional well-being among adolescents [61] and perceived stress [21]. Although some researchers have found negative health consequences of sedentary behavior, the association between mental health and sedentary behavior is inconsistent [62]. A systematic review showed that there was limited available evidence demonstrating that student sedentary behaviors are significantly related to mental health [63]. Students participating in physical activity during school time may present better physical, psychosocial, and cognitive health compared to those who were sedentary during any school physical activity opportunities [64]. This study demonstrates that physical activity significantly affects sedentary behavior and screen time and reduces bully behavior. Chinese high school students face their greatest challenge in the college entrance examination. This study showed that increased sedentary time results in a significant decrease of physical activity as well as physical fitness, which reflects the reality of school-based physical activity. Increasing physical activity can reduce sedentary behaviors, such as watching TV and video gaming [65, 66]. Physical activity can be used to design effective intervention programs to change unhealthy habits and risk behaviors of adolescents and promote their physical health [67]. Some studies demonstrate that adolescents who are actively encouraged to participate in more exercise will reduce screen time, develop friendships and increase their emotional well-being [68]. Physical activity has protective and indirect effects on smoking and alcohol use in adolescents [29, 69]. Some research indicates that educating adolescents to avoid using alcohol and to maintain proper healthy diet habits does not have a more significant impact on fitness than does encouraging them to participate in sufficient physical activity [70].

### Strength and limitation

There are several strengths and weaknesses in this study. This study is the first time to focus on clusters of risk behaviors and factors that may be associated with mental health and physical activity in Chinese adolescents. Previous studies have investigated subcategories of risk behaviors, but this study explored the association between a more comprehensive set of behaviors and mental health. Integrating clusters construction and factor analyses were utilized in this study. The strong factor loadings and significant association were identified. Validated survey instruments were utilized to collect data on physical activity, risk behaviors and mental health. The response rate of 95.8% exist not response bias. The limitation of the current study is cross-sectional investigation, it was not possible to define the temporal relationship between cause and consequence, but our approach allowed us to assess relationship between risk behavior, mental health and physical activity. However, the relationship needs to be further verified by behavior intervention experiments and physiological mechanisms. In addition, this study chose a sample of high school students from the Shandong province in China, thus the results may not be generalizable to all of China. The relative lack of sociodemographic variables might also limit the comprehensiveness of some outcomes.

## Conclusion

This study finds that a specific behavior cluster has significant impact on mental health and physical activity among adolescents. This study shows that integrating risk behaviors cluster with factors can be employed to target high-risk adolescents who have poor physical and psychosocial health. The findings of this study suggest that more effective and feasible school intervention programs can be designed to promote adolescent health-related behavior in terms of those pathways. However, examination of the clustering of risk behaviors should further explore determinants across diverse types of health behaviors for adolescent intervention programs.

## Data Availability

The datasets during and/or analysed during the current study available from the corresponding author on reasonable request.
